# Engineering Efficacy and Accuracy in the Optical Printing of Dielectric Nanocrystals

**DOI:** 10.1021/acs.nanolett.5c06116

**Published:** 2026-04-02

**Authors:** Brandon Reynolds, Matthew J. Crane

**Affiliations:** † Department of Chemical and Biological Engineering, 3557 Colorado School of Mines, Golden, Colorado 80401, United States

**Keywords:** Optical printing, dielectric, nanocrystal, nanofabrication, directed assembly, photothermal

## Abstract

Accurate single nanocrystal positioning tools are critical for emerging quantum and photonic technologies. Optical printing provides a platform to realize this positioning, but we need to understand the roles that solvent and surface functionalization play in controlling positional error, particularly for dielectric nanocrystals. Here, we characterize the impact of solvent and surface functionalization on nonresonant optical printing accuracy and efficacy, demonstrating accuracies of 50 nm. Changing solvent, nanocrystal concentration, and surface functionalization influences the number of attempts required to print nanocrystals, which we find is connected to positional error. Adding electrolytes to modify the interfacial DLVO potential reveals a unique regime where high irradiances minimize positional error while maximizing efficacy. Multiphysics simulations suggest that this regime results from photothermal effects and gradient forces. We validate these simulations by showing lower positional errors from substrates with higher absorption coefficients. These results will guide optical printing procedures for single nanocrystal positioning applications.

## Graphical abstract

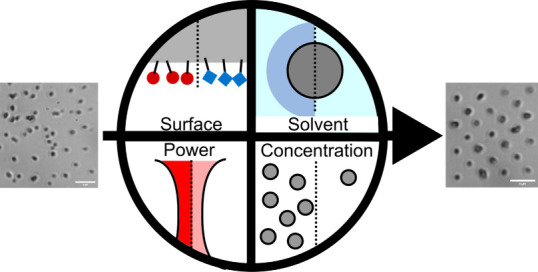

Patterning techniques to control the orientation and position of single colloidal nanocrystals promise to enable critical technologies, such as quantum communication,^1–5^ physical unclonable functions,^6–8^ and optical data storage.^9–14^ Current approaches to single-particle patterning range from deterministic strategies such as photothermal driven printing,^15–18^ electron beam lithography,^19^ lithographically defined templating,^20–24^ or pick-and-place techniques^25–29^ to stochastic approaches like spin coating or drop casting^30–32^ on substrates with redundantly patterned targets. However, simultaneously achieving high spatial precision and control of orientation using these methods is difficult. Optical printing is a technique that can address this challenge by using the scattering and gradient forces from a focused laser beam to move particles to a target location and press them onto a surface, where the van der Waals forces secure them in place. The polarization of the laser can orient particles,^33–37^ and the high gradient forces enable excellent spatial precision, achieving sub-100 nm accuracies in some instances.^38^

Stefani and co-workers^39^ laid the foundation for understanding accuracy in 60 nm diameter gold nanocrystals, noting that the key to accuracy is balancing the axial printing force and the repulsive surface interaction. For example, in aqueous systems, the surface interaction is mediated via DLVO forces, which balances the attractive van der Waals force with a repulsive electric double layer force. They concluded that optical printing accuracy is maximized at lower irradiances, when the optical scattering force can only overcome opposing surface forces in a small region. However, low irradiances also increase the print time, limiting simultaneously optimized accuracy and high-speed operation. Most of the work on optical printing fundamentals has utilized coinage metal nanocrystals such as gold or silver,^36,38–42^ rather than semiconductor or dielectric nanocrystals, due to the high polarizability of the metals that maximizes the force of radiation pressure. Similarly, the optical printing of dielectric nanocrystals has only been studied under resonant conditions.^43,44^ As a consequence, much of our understanding is rooted in metal plasmonic nanocrystals and in resonant optical printing conditions. Yet, many of the most enticing patterning applications involve nonmetallic nanocrystals, such as quantum communication applications, which rely on semiconductor nanocrystals that do not exhibit resonances at practical wavelengths.

Here, we investigate how operating conditions influence the optical printing of semiconductor TiO_2_ nanocrystals onto substrates with various surface functionalization, solvents, and nanocrystal concentrations under nonresonant conditions. We demonstrate the robust optical printing of nanocrystals with accuracies down to 50 nm under a range of conditions. By analyzing the impact of these variables on optical printing accuracy and efficacy—i.e., how many attempts are required to bind a nanocrystal to the surface—we find that efficacy is primarily controlled by surface tension and can be modulated by controlling the surface via functionalization, solvent, and nanocrystal concentration. These experiments also reveal correlations between efficacy and accuracy, that depend sensitively on the magnitude of interfacial forces and irradiance. At high electrolyte concentrations, printing accuracy is improved at lower irradiances consistent with Gargiulo et al.;^39^ whereas, at low electrolyte concentrations, printing accuracy is improved by high irradiances. This previously unobserved regime in low electrolyte concentrations enables the simultaneous improvement of accuracy and efficacy. Multiphysics simulations show that these regimes are due to a combination of thermal effects and the momentum of the nanocrystal. Based on these observations, we establish a framework to control efficacy and accuracy in optical printing, which will guide the use of optical printing as a single nanocrystal printing tool.

Figure 1 details the process of optical printing for a spherical nanoparticle. A laser is focused onto a substrate at the target position for nanoparticle printing. Optical scattering and gradient forces exerted on the nanoparticle by the laser move the particle toward the focal point. Along this path, the surface and viscous effects also exert forces on the particle. These surface forces are typically repulsive. When the optical forces exceed the repulsive surface forces, the nanoparticle can contact the surface after which van der Waals forces between the substrate and nanoparticle lock the particle in place. There are two key parameters that govern the ability to create patterns of printed particles. The first is efficacy, shown on the left side of Figure 1, which is the number of attempts required before a particle prints. Particle adhesion behavior is a standard performance metric for many nanocrystal deposition techniques, not just optical printing.^45,46^ An example of this experiment is shown in Figure 1, where a 300 nm diameter TiO_2_ nanocrystal in water at an irradiance of 3.1 MW/cm^2^ required 58 attempts to print on clean glass. We quantify efficacy by computing the average of the required attempts to print, ⟨N_att_⟩. The second parameter is accuracy, shown on the right side of Figure 1, which is the distance between the printed position relative to the target printed position. For many applications, precise deposition of nanocrystals is critical, as has been previously studied in optical printing^38,39^ and in other deposition techniques.^45,47^ An example of this experiment is shown on the right side of Figure 1 with a 5 × 5 printed array of 300 nm diameter TiO_2_ nanocrystals spaced 4 μm apart. By fitting the measured positions of each particle to a 5 × 5 grid with 4-μm spacing between particles, we extract the error in printed particle position as a standard deviation, σ_position_. We note that 300 nm diameter TiO_2_ nanocrystals were selected to avoid Mie resonances that complicate optical force calculations and due to their high refractive index, which enhances optical forces and matches many promising colloidal materials for quantum applications.

**Figure 1:**
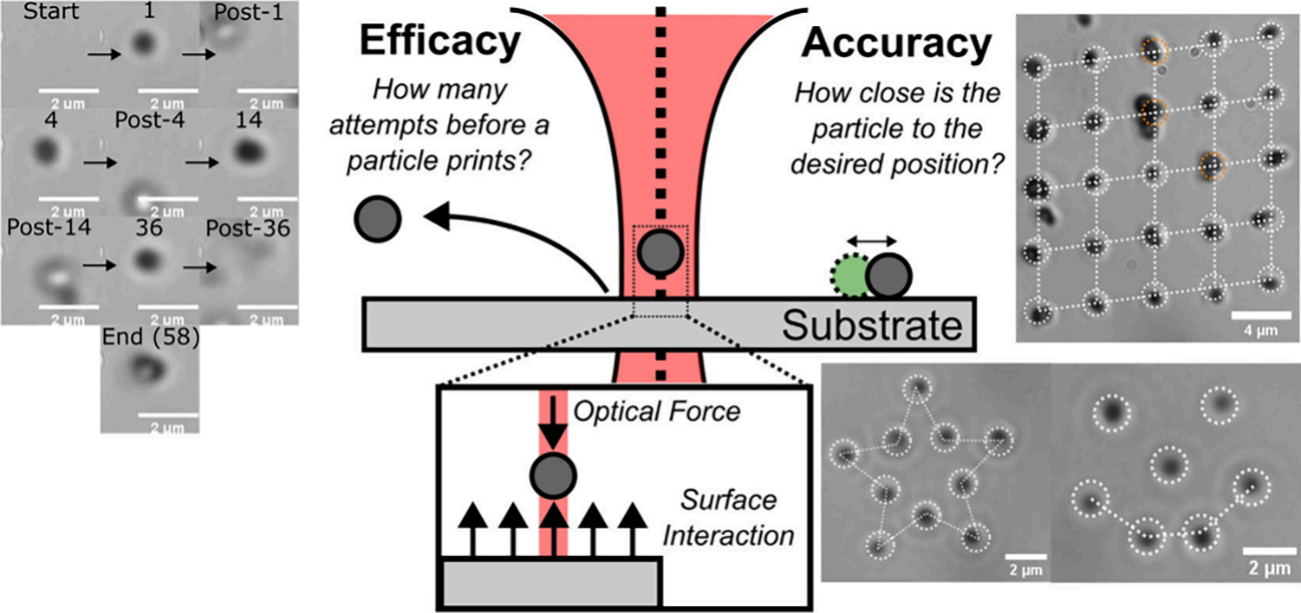
Schematic for optical printing, efficacy, and accuracy. The center illustrates the optical printer, which relies on optical scattering and gradient forces to direct a colloidal particle to a desired location on a substrate. These optical forces must overcome repulsive substrate forces to print. The left side of the figure shows printing efficacy, which is a measure of how many attempts are necessary before a particle prints. A typical experiment is shown on the very left. Before the laser is turned on there is no particle on the surface. The laser is then exposed to the sample (1 s on, 0.5 s off) in a single print attempt. When the laser is exposed to the chamber, a particle is pushed to the surface; when the laser is off, the particle diffuses if it is not printed or remains if it is printed. The number of times the laser was exposed to the surface before a print event occurs is the print “efficacy”. Accuracy is shown on the right side. Here, accuracy is defined as a measure of how close the printed particle’s position is to the desired print position. Microscope images show examples of realistic printing accuracy experiments, where the particles were printed into a 5 × 5 square lattice with a desired interparticle distance of 4 μm. To demonstrate the variety of patterns that can be printed, a smiley face and star were printed. White dashed lines are present to guide the eye to the printed pattern, and white dashed circles are present to identify desired particle positions. Orange dashed circles represent multiparticle printing events, which are also included in accuracy measurements (see Figure S2).

Figure 2A shows ⟨N_att_⟩ of 300 nm diameter TiO_2_ nanocrystals as a function of irradiance for a variety of different experimental conditions. When printing onto a clean silica surface, the average number of attempts to print decreases as the irradiance increases. The solid lines are fits proportional to the inverse of irradiance squared, which reflects the momentum of the nanoparticle (*vide infra*). The ⟨N_att_⟩ varies depending on the solvent, concentration of nanocrystals, and surface treatment. The ⟨N_att_⟩ is larger for ethanol compared to water, and increasing the nanoparticle concentration lowers the ⟨N_att_⟩. This irradiance dependence disappears when the surface is treated with APTES.

**Figure 2:**
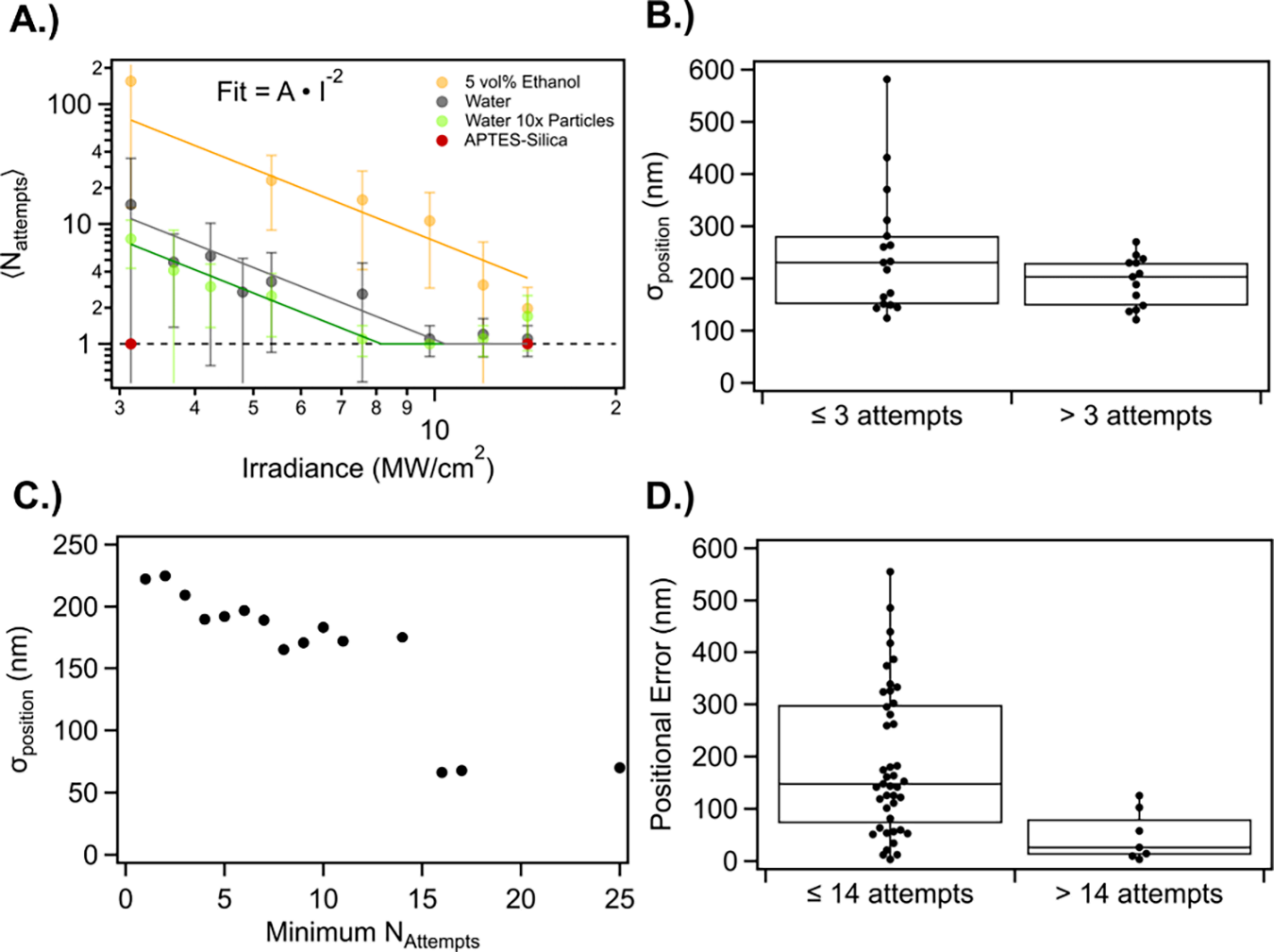
(A) A log–log plot of ⟨N_att_⟩ for TiO_2_ nanocrystals in different solvents and at different irradiances. Each data point corresponds to at least ten separate particle prints, and the error bars are the standard deviation of the number of required attempts for those ten prints. Error bars show the standard deviations. The solid lines are fit lines using an inverse squared relationship with irradiance. The dashed line is for required print events of 1, the minimum possible value. (B) Box and whisker plot correlations between ⟨N_att_⟩ ≤ 3 and ⟨N_att_⟩ > 3 and σ_position_ for the printing of 300 nm diameter TiO_2_ nanocrystals in different aqueous solutions onto glass substrates. The solutions ranged from 0 to 17.1 mM NaCl in water and also include 5 vol % ethanol in water, and 0.2 g/mL glucose in water. (C) The σ_position_ for 50 printed 300 nm diameter TiO_2_ nanocrystals on glass with an irradiance of 5.4 MW/cm^2^ in water binned by the minimum number of attempts required to print. The minimum number of attempts here includes all particles that required at least that number of attempts to print onto the surface. (D) Box and whisker plot comparing individual positional error for particles N_att_ ≤ 14 vs N_att_ > 14. All experiments were performed with a 976 nm laser focused through a NA = 1.3, 100x objective.

We interpret the changes in ⟨N_att_⟩ with solvent, surface, and particle concentration to reflect the different forces involved throughout printing. For example, the addition of ethanol to water decreases the surface tension of the solvent relative to the substrate,^48^ which reduces the force of adhesion (see SI for more detail). This change in surface tension in 5 vol % ethanol creates a larger barrier that the optical forces must overcome compared to water, increasing the ⟨N_att_⟩. Consistent with this interpretation, APTES functionalization of the glass surface increases the force of adhesion for the particle to the surface, lowering the barrier for printing such that all particles are printed on the first attempt (i.e., ⟨N_att_⟩ = 1). Conversely, increasing the concentration of TiO_2_ nanocrystals enhances local fluid convection due to drag forces from the moving nanoparticle by a factor of 10. Such convection effects have been observed previously in optical trapping experiments.^49^ As a result, nanocrystals in higher concentration solutions experience greater velocities and kinetic energies at identical irradiances throughout the printing process. As detailed below, we assign the decrease in ⟨N_att_⟩ at higher particle concentrations as an impact of the higher average particle kinetic energy, which reduces the required optical force to print. Indeed, this assignment is consistent with the *I* ^–2^ dependence of ⟨N_att_⟩, where the nanocrystal is at its terminal velocity throughout printing. This positive relationship between particle sticking and momentum has been previously noted in spray deposition techniques.^50–52^

The ⟨N_att_⟩ is also tied to σ_position_. Figure 2B compares the σ_position_ for low ⟨N_att_⟩ (≤3 attempts) and high ⟨N_att_⟩ (>3 attempts). While the average σ_position_ was only slightly reduced for high ⟨N_att_⟩ cases compared to low ⟨N_att_⟩ cases, there was a large difference in their deviations. Low ⟨N_att_⟩ cases had a large spread in σ_position_, visualized by the large range and extended quartiles of the box plot. In comparison, high ⟨N_att_⟩ cases had a smaller spread in σ_position_ and did not depend on experimental conditions like salt concentration, particle concentration, or irradiance. Figure 2C and D also shows this connection between accuracy and efficacy by addressing each particle in the printed array individually rather than as a whole. In Figure 2C, we observed a negative trend with σ_position_ as a function of minimum required attempts to print. Figure 2D shows the positional error for individual particles that required more than 14 attempts to print vs fewer than 14 attempts to print. Unlike particle error binned at ⟨N_att_⟩ = 3, the box and whisker plot around ⟨N_att_⟩ = 14 indicates a drastically reduced σ_position_ for nanocrystals printed with more than 14 attempts.

We interpret this difference in mean σ_position_ for different ⟨N_att_⟩ as a change in the repulsive forces for an individual printing event, consistent with the previous results of Gargiulo et al.^39^ In low ⟨N_att_⟩ conditions, the repulsive forces are lower, which enables the particle to print over a larger area along more possible trajectories, increasing the σ_position_. Conversely, at high ⟨N_att_⟩ conditions, the repulsive force is greater, and there are fewer trajectories that lead to printing. Spatially varying surface energies are likely due to adsorbates or variations in silanol groups, roughness, or composition.^53–55^

To investigate the impact of colloidal forces and kinetic energy on printing, we next explore the dependence of σ_position_ on solvent and irradiance. Figure 3A shows the ratio of σ_position_ for arrays printed with 14.3 MW/cm^2^ to arrays printed with 3.1 MW/cm^2^ as a function of NaCl concentration. The addition of NaCl decreases the Debye length modifying the profile and amplitude of the electric double layer to oppose printing in water (Figure S5).^56,57^ As the NaCl concentration increased, the ratio grew from 0.48 to 1.36. At low NaCl concentrations, particles printed at high irradiances exhibited lower σ_position_ than particles printed at low irradiances; while at high NaCl concentrations, low irradiances led to lower σ_position_ compared to high irradiances. This σ_position_ dependence on NaCl concentration supports the existence of an opposing force proposed by Gargiulo et al.^39^ However, unlike these previous reports on ∼60 nm diameter Au and Ag nanocrystals in water, which found that increasing irradiance increased σ_position_, we instead observe that increasing irradiance reduces σ_position_ at low NaCl concentrations. Increasing the concentration of NaCl reverses this behavior, eventually causing a reduction in σ_position_ at low irradiances relative to high irradiances, consistent with previous results.^38,39^ Figure 3B plots σ_position_ for 300 nm diameter TiO_2_ nanocrystals at two different irradiances on APTES-functionalized glass, which reduces the forces that oppose printing. Again, higher irradiances reduced σ_position_ compared to lower irradiances.

**Figure 3:**
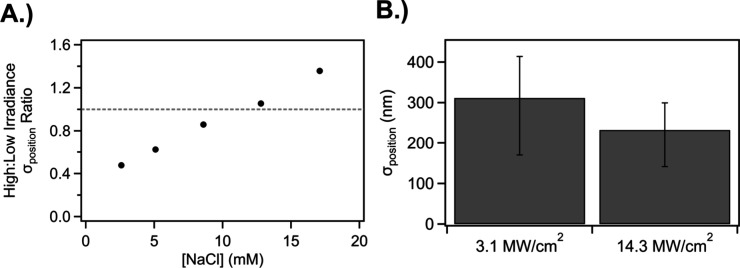
Increasing the laser irradiance can improve σ_position_ under specific conditions. (A) The ratio of σ_position_ for high irradiance (14.3 MW/cm^2^) to low irradiance (3.1 MW/cm^2^) as a function of salt concentration. All experiments here were performed for 300 nm diameter TiO_2_ nanocrystals in water with varying concentrations of salt, with a cleaned glass slide as a substrate. All experiments shown here were nanocrystals that required fewer than 3 attempts to print on average, which we categorize as low ⟨N_att_⟩. The 17.1 mM is an average of two separate experiments. The gray dashed line is at a ratio of 1.0. (B) A comparison of σ_position_ for high and low irradiances, for printing 300 nm diameter TiO_2_ nanocrystals in pure water on APTES-functionalized glass slides. Error bars here are from a statistical bootstrapping analysis to give an idea of the variation, an explanation of this analysis is given in the Supporting Information. All experiments shown were performed with a 976 nm laser focused through a NA = 1.3, 100x objective.

We constructed a range of models to explain the irradiance and salt dependence on σ_position_, which reveal that optical and surface interaction forces alone cannot explain the behavior of reduced σ_position_ at higher power. Instead, it is necessary to include the impact of elevated temperature. Under the high irradiances of the NIR laser, the solvent and surface may heat, and this elevated temperature distribution impacts the particle trajectory in different ways. The local viscosity is a strong function of temperature and will decrease under elevated temperature,^58^ changing the drag force. In addition, thermophoretic forces^59–63^ and thermally driven convection forces become relevant.^59,64,65^ Upon increasing the irradiance, the local temperature will be higher, changing the magnitude of these effects.

To understand the impact of these different phenomena, we constructed a multiphysics model that incorporates these optical, colloidal, and kinetic forces as well as temperature-dependent properties. Details of this model are shown in the SI, including COMSOL modeling (Figure S3). Figure 4A shows a colormap of the thermally induced force field due only to thermophoretic and convective forces. The photothermal heating effects are simulated to originate from both the solvent and surface, consistent with our experimental observations. Within 3 μm of the surface, the thermal force field lies almost entirely in the radial direction away from the laser focus. Beyond 3 μm, the axial and radial components of the force field are of similar magnitude, with the radial component pointing away from the laser focus and axial component pointing toward the laser focus.

**Figure 4:**
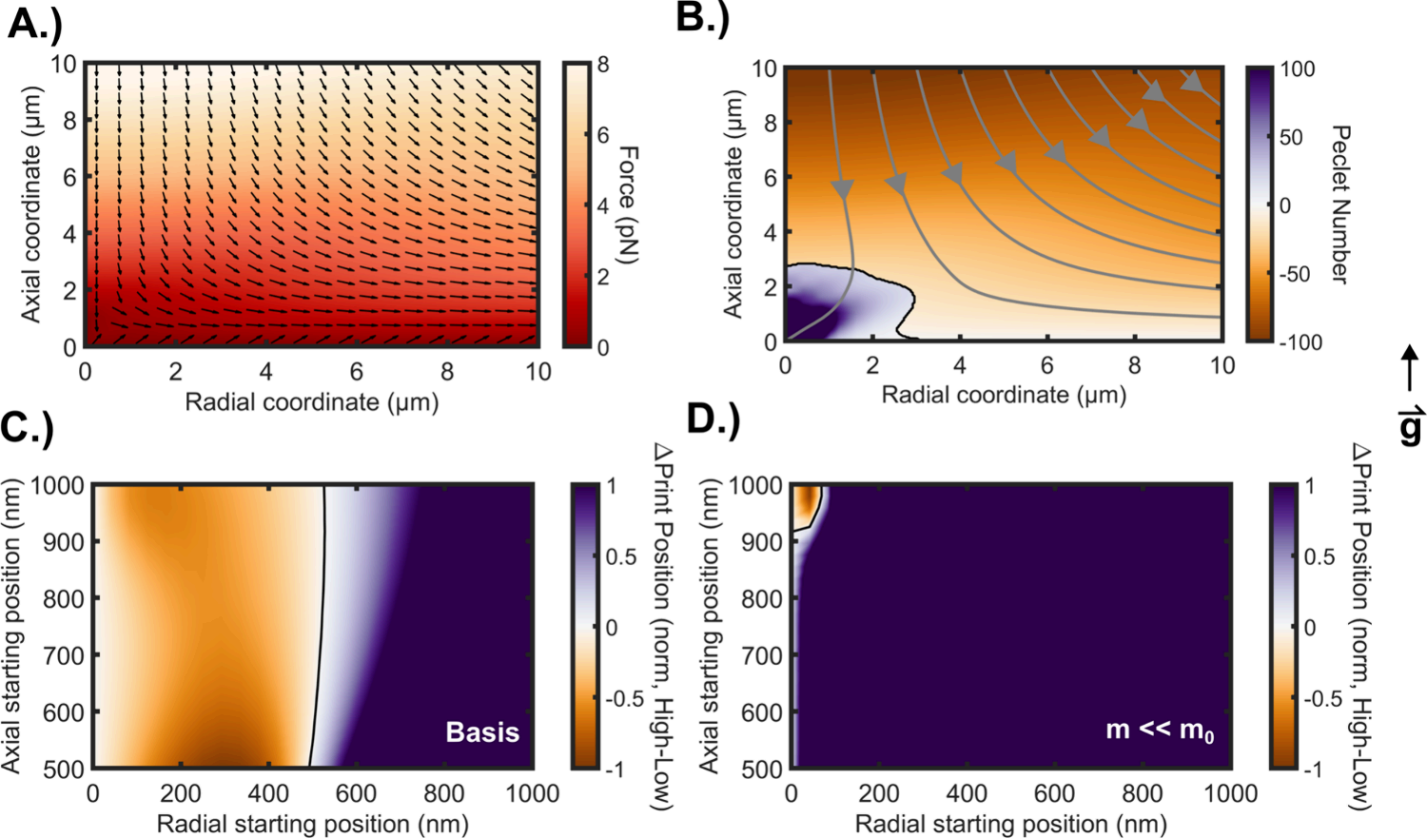
Impact of heating on optical printing. (A) Colormap showing the computed thermally induced force field that particles experience near the laser focus due to convection and thermophoretic forces. The arrows represent the direction of the force at each location. (B) Local Peclet (Pe) number during optical printing under heating conditions, with streamlines shown in gray. Negative and positive Pe numbers represent regions where the thermal forces dominate and where the optical forces dominate, respectively. The arrows show the direction of the force on the particle. (C, D) Surface plots of the normalized change in predicted print location relative to the desired print location for particles starting from different locations. The color represents the difference between the final printed position for a particle irradiated with high power (112 mW) subtracted from the final printed position of a particle irradiated with low power (24.6 mW). Kinematic modeling of particle trajectories in a typical printing event including DLVO surface interactions and kinetic energy under elevated temperature, using the simulated temperature distribution and thermophoretic force, with the experimental values for convection velocities. (C) Simulation for 330 nm diameter TiO_2_ nanocrystals in water. (D) Same simulation as in panel C but assuming particles immediately reach terminal velocity, thereby neglecting the kinetic energy of the particles. All temperature distributions and convective velocity distributions simulated in COMSOL, and convective velocity was scaled to match experimental observations (see Supporting Information for more detail). Simulation conditions and procedure are discussed further in the manuscript and in the Supporting Information. In all panels, the radius = 0 and axial = 0 is the laser focus and desired printing location.

To understand which forces dominate in different regions, we conducted a Peclet (Pe) number analysis, which compares the ratio of optical or thermal advection to diffusion. Figure 4B shows the simulated Peclet number for a 330 nm diameter TiO_2_ nanocrystal in water. As described above, while the magnitude of the force depends strongly on the thermal profile, the direction of the force field is relatively invariant. Gray lines represent streamlines that a particle would follow under the thermal and optical forces. The plot includes two Peclet numbers, representing the optical and thermal forces. For these simulations, Peclet number is calculated using eq 1 ^39^

$$\mathrm{Pe}=\frac{r_{p}F}{3k_{B}T}$$ 1

where *r*_*p*_ is the radius of the particle, *F* is the optical or thermal force acting on the particle, *k*_*B*_ is the Boltzmann constant, and *T* is the local temperature. Based on the magnitude of Pe numbers, particles close to the surface are always dominated by either thermal or optical convection, rather than diffusion. Due to these thermal and optical forces, only certain trajectories lead to printing. For example, for particles starting 10 μm from the surface, only those with radial positions <2 μm are printed. Initially, the printing force for these particles is due to thermal effects (Pe < 0) before optical forces dominate (Pe > 0). In comparison, the trajectory of particles at radial positions >2 μm never leads to a region where optical forces dominate, and these nanoparticles are rejected due to thermal forces. As a result of this thermal force distribution, only axial trajectories are favored for printing.

Figure 4C shows the normalized difference in printed position between high power and low power cases for 330 nm diameter TiO_2_ nanocrystals in water as a function of starting position—i.e., negative values indicate regions where particles are printed more accurately with lower standard deviation. These simulations include kinetic energy and DLVO forces as surface interactions. Radial starting positions <500 nm show a reduction in σ_position_ for high power compared to low power printing, consistent with our experimental observations. Under these conditions, starting positions radially farther from the laser axis have reduced σ_position_ at low powers compared to high powers. A contour line indicates the location where σ_position_ switches from being reduced to being elevated by high powers.

These simulations reveal reduced local viscosity minimizes σ_position_ in all experimental conditions via its impact on kinetic energy. When viscosity is lower, the drag force acting on the particle is reduced, increasing the terminal velocity and therefore the kinetic energy of the particle. Once the particle comes in range of the DLVO surface interactions, the particle begins to decelerate. Due to the non-negligible mass of these particles, rather than always being at local terminal velocity, particles decelerate as they move through the DLVO force field. As a result, particles with higher radial kinetic energy move closer to the optical axis than particles with lower radial kinetic energy. In the high irradiance case, higher heating reduces the local viscosity more than low irradiances, leading to an increase in radial momentum and thus, a reduction in σ_position_. If the particle starts further away from the optical axis, the radial optical force is weaker, and this kinetic energy effect is diminished. Removing the temperature dependence of viscosity also removes this kinetic energy effect (see Figure S4).

As seen in Figure 4C, however, this kinetic energy effect is only relevant within ∼500 nm of the optical axis. To explain the reduced σ_position_ at high irradiances, there must be an additional power-dependent effect that works to reject particles that start from further away. This thermal rejection phenomena is driven by convection and thermophoretic forces. Given the force profile in Figure 4A, particles that start at radially far distances are pushed away from the laser by these thermal forces and not printed. In contrast, on-axis particles experience an axial force pointing toward the surface which avoids convection while shuttling particles to the print location. The combination of these phenomena leads to a power-dependent skew that favors on-axis particles that are printed more accurately due to their radial momentum. The combination of these convective and kinetic energy effects reduces σ_position_ for high irradiances.

Figure 4D shows the same simulations as Figure 4C but neglects kinetic energy to emphasize its importance. Without kinetic energy, the high irradiance-induced reduction in σ_position_ should vanish. Indeed, as visualized in Figure 4D, without kinetic energy, the majority of the simulated region exhibits an elevated σ_position_ at high irradiances. Only starting positions close to the optical axis have reduced σ_position_ at higher irradiance. These results indicate that small nanocrystals without significant mass will always be printed with elevated σ_position_ at higher irradiances, consistent with previous observations.

A final important feature of our experimental results is that at [NaCl] > 13 mM, higher irradiances increase σ_position_ compared to lower irradiances, whereas at low salt concentrations, this behavior inverts. At all tested concentrations, the refractive index is virtually constant, and the salt concentration primarily changes DLVO interactions and to a lesser extent, the Soret coefficient. As salt concentration increases, the DLVO electrostatic interactions are compressed into a smaller volume, increasing the maximum surface repulsive force (see Figure S5). Because the DLVO field is compressed into a smaller region, particles with sufficient axial kinetic energy can traverse the opposing DLVO force field without significant deceleration. As discussed above, the radial kinetic energy during this DLVO-related deceleration provides the greatest benefit to σ_*position*_. Accordingly, particles that spend a longer time traversing the repulsive domain experience the greatest benefit to accuracy. Under high irradiances, particles have a high axial kinetic energy and spend a shorter time in the DLVO field, which reduces their benefit. In low irradiances, particles spend a longer time in the DLVO field, allowing for the radial kinetic energy to bring the particle closer to the optical axis. As a result, at high salt concentrations, low irradiances lead to lower σ_position_ than high irradiances, which can be seen in Figure S6.

To test this model, we printed onto a 10% Sn^4+^:In_2_O_3_ (ITO) surface, which has a higher absorption coefficient at the trapping laser wavelength compared to glass (α_*ITO*_ = 1500 cm^–1^ *vs α*_*glass*_ = 0.6 cm^–1^),^66,67^ and therefore enhances heating. Figure 5A shows a comparison of σ_position_ for 300 nm diameter TiO_2_ nanocrystals in water on cleaned silica vs on ITO. An irradiance of 1.35 MW cm^–2^ was used because higher irradiances led to bubble formation, confirming the enhanced heating. The ITO-coated glass shows a more than 35% reduction in σ_position_ compared to that of the bare glass at the same irradiance, consistent with our model predictions that thermal effects lead to a reduced σ_position_. To ensure the results were directly comparable, 2.6 mM NaCl water was used on the glass to match the ⟨N_att_⟩. It should be noted that this elevated salt concentration would only reduce the σ_position_ compared to that of pure water, and therefore the effect seen here is not a result of the media. In addition, the contact angle of these solutions on the substrate was measured to ensure that surface tension effects could be neglected (see Figure S7). Finally, we note that ITO has a lower magnitude zeta potential than SiO_2_, which is expected to cause an increase in σ_position_.^68–70^ Indeed, Figure S8 shows that decreasing zeta potential by modulating pH increases σ_position_. Despite these factors, we observe an improvement of σ_position_, consistent with our model’s predictions, due to photothermal effects.

**Figure 5:**
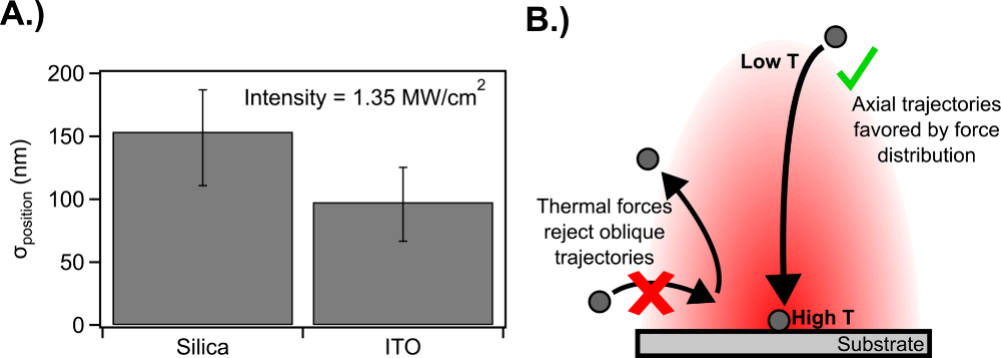
Impact of photothermal heating on σ_position_. (A) σ_position_ of printing 300 nm TiO_2_ nanocrystals in 2.6 mM NaCl in water on glass vs particles in pure water on ITO-coated glass. (B) Schematic depicting the optical printing process under heating conditions for particles in initially oblique locations compared to axial locations. All experiments shown were performed with a 976 nm laser traveling through a NA = 1.3, 100x objective at an irradiance of 1.35 MW/cm^2^.

Figure 5B shows a schematic depicting the impact of photothermal heating on σ_position_ for particles with appreciable momentum. Particles are randomly dispersed in the solution and will diffuse through the solution until the optical forces begin to attract the particle. Whether these forces are predominately optical or thermal depends on the local Peclet number. Thermal forces will act to repel the particle away from the laser focus, but the thermal force acting on the particle depends on where the particle starts. If the particle approaches from an oblique streamline, the thermal forces ultimately exceed the optical forces, causing the particle to be rejected. In contrast, if the particle approaches from an axial trajectory, the optical forces exceed the thermal forces, leading to contact with the surface.

Our data indicate a new previously unobserved “inverted” regime where optical printing accuracy is improved at high irradiances. Compared to previous studies from Stefani and co-workers,^42^ we interpret the new regime as being due to the larger mass of our particles and photothermal effects. However, there are a few additional key differences. First, while we use an inverted microscope and therefore print on the top surface of a chamber, previous reports have printed on the bottom surface with an upright microscope. This geometry change creates different thermal force profiles due to the different orientation of the laser relative to gravity. Second, our laser wavelength is nonresonant with the particles, while previous optical printing experiments were resonant with the particle. As has been noted, when nonresonant conditions are used, the original framework does not match the experimental results, and power-accuracy relationships can be nontrivial.^71^ Our data suggest that this discrepancy may be explained by momentum and thermal effects. Leveraging these effects, we demonstrate optical printing with accuracies of 50 nm, lower than those reported for nonresonant optical printing of metal nanocrystals (∼100 nm)^71^ or optothermophoretic printing (∼200 nm; ∼80 nm with correction), which relies on thermal mechanisms for attachment.^73^

Our observations also inform a new framework for optical printing. Tuning the surface, solvent, and irradiance as well as the particle geometry and composition can control both σ_position_ and ⟨N_att_⟩. In applications where printing efficacy is critical, increasing irradiance or surface tension, e.g. by judicious choice of solvent, substrate, or surface functionalization, will lower ⟨N_att_⟩. Similarly, increasing the number of nanocrystals in solution and modifying repulsive forces via salts will lower ⟨N_att_⟩. Conversely, low irradiance, surface tension, and particle concentration will avoid nonspecific printing. Maximizing accuracy in aqueous solvents depends on the electrolyte concentration. In high electrolyte concentrations, low irradiances boost accuracy (i.e., minimize σ_position_); in contrast, in low electrolyte concentrations, high irradiances boost accuracy. Increasing optical forces by changing particle size also offer an approach to modulate accuracy. Finally, local temperature gradients on the surface are beneficial to lowering σ_position_ in an inverted microscope. Selecting substrates, solvents, and particles to maximize these gradients can thus lower σ_position_. Notably, these results indicate a unique region in which both σ_position_ and ⟨N_att_⟩ can be minimized. However, as noted above, this framework may depend on optical printer geometry. While we do not test organic solvents here, we anticipate that these guidelines for ⟨N_att_⟩ and σ_position_ will be applicable. For example, depletion forces may provide a mechanism to control these parameters.^74^ Similarly, while we study 300 nm diameter TiO_2_ nanocrystals, we anticipate that this framework will also apply to different materials, where tuning the ratio of optical forces to repulsive forces via particle properties provides a pathway to modify efficacy and accuracy.

In summary, we have demonstrated robust optical printing under nonresonant conditions and identified a new regime of optical printing in which both accuracy and efficacy can be optimized. By measuring ⟨N_att_⟩ and σ_position_ in different conditions, we observed an inversion in which high irradiances led to improved accuracies. These results are consistent with multiphysics models in which thermal convection and thermophoretic forces enhanced printing accuracy via the kinetic energy of optically trapped particles. We anticipate that these results will provide critical insight into optical printing-based nanocrystal fabrication processes and guide the use of optical printing as a highly accurate, single nanocrystal patterning tool.

## Supplementary Material



## Notes

Experimental details including substrate and nanocrystal preparation, simulation details including modeling of thermal phenomena and complete set of equations used in Multiphysics model, discussion of bootstrapping analysis, discussion of adhesion force and its relation to efficacy, simulation results excluding thermal dependence of viscosity, DLVO simulations as a function of electrolyte concentration and distance to surface, simulation results under elevated electrolyte concentration, contact angle measurements for ITO and silica glass, diagram of experimental optical printer setup (PDF)
