# Supplementary material for: Engineering Efficacy and Accuracy in the Optical Printing of Dielectric Nanocrystals
Source: Nano Lett. 2026 Apr 2;26(14):4580–8. doi: 10.1021/acs.nanolett.5c06116 (PMC13088359; doi:10.1021/acs.nanolett.5c06116)
Supplement: Supplementary file 1 [file nl5c06116_si_001.pdf]

Supporting Information for Engineering Efficacy and Accuracy in the Optical Printing of Dielectric Nanocrystals

Brandon J. Reynolds,[†] Matthew J. Crane[†,*]
[†]*Department of Chemical and Biological Engineering, Colorado School of Mines, Golden, CO 80401*
*Email: matthewcrane@mines.edu

Experimental section:

Sample preparation: The substrates used in these experiments were 32 mm x 25 mm glass substrates or ITO substrates. To normalize the surface across experiments, substrates were sequentially sonicated in a soapy water solution, acetone (Pharmco product 329000000), and isopropanol (Pharmco product 231000099). Substrates were sealed in a container while the particle solution was prepared, no more than 30 minutes. For APTES-functionalized surfaces, glass surfaces were cleaned and then immersed in a container with 14 mL toluene (Sigma-Aldrich product 244511) and 0.8 mL APTES (Sigma-Aldrich product 440140) for 18 hours. After 18 hours, the substrates were removed and sonicated in ethanol (Pharmco product 111000200) for 15 minutes, dried, sonicated again for 15 minutes, dried, and then sealed in a container while the particle solution was prepared, no more than 30 minutes.

The standard particle solution was prepared by mixing 1 µL of 300 nm TiO_2 10 wt% in water solution (Sigma-Aldrich product number 914177) with 1 mL MilliQ water to create an initial 1000x dilution, and then 100 µL of that solution was mixed into 0.9 mL MilliQ water to create an additional 10x dilution. This dilution gives an approximate particle concentration of 170,000 particles per mL, which led to a particle being printed within 30 seconds while not being so concentrated as to have multi-print events dominate. Variations on this standard procedure are

detailed as follows. For the 10x particle concentration (Figure 2A) the 10x dilution step was not performed. For the NaCl based solutions, the molarities tested were 2.6 mM, 5.1 mM, 8.6 mM, 12.8 mM, and 17.1 mM, based on the final 1 mL solution volume. For the 5 vol% ethanol data set (Figure 2A), the particles were added to 50 µL ethanol with 850 µL MilliQ water during the 10x dilution step, instead of 900 µL MilliQ water. For Figure 2B, a point with 200 mg/mL glucose was used, which was prepared such that the final concentration was 200 mg/mL. The particle solution was sonicated for 15 minutes prior to being used. To prepare printing chambers, a Grace Biolabs SecureSeal spacer (product number 654002) was adhered to the prepared substrate, and a glass #0 coverslip was wiped clean with methanol to act as the cover for the chamber. After sonication of the particles, ~ 300 µL of particle solution was added in the center of the chamber, and the coverslip was gently pressed on top, ejecting excess liquid and creating a sealed chamber with no visible air bubbles. The chamber was immediately used for printing.

Optical printer: A homebuilt optical printer was utilized for optical printing experiments as described previously.[1] This optical printer utilizes a 976 nm laser with irradiances ranging from 1.35 MW/cm^2 to 14.3 MW/cm^2 measured after the objective with a spot size of approximately 1 µm. The objective used for these experiments is a Nikon 100x oil-immersion objective with NA = 1.3. A Thorlabs camera (CS165MU1) is used to image the particles in a brightfield configuration. The optical printer utilizes a piezo stage (Thorlabs NanoMax 300) to move particles to precise positions for printing.

Simulation: Simulations were performed utilizing Matlab, incorporating custom code with code from the Optical Tweezers Toolbox in Matlab.[2] This simulation incorporated thermal forces including thermophoresis and thermal convection forces, as well as kinetic energy of particles, drag forces, Faxen's correction, and more, detailed in a separate section in the Supporting

Information. To gather the local temperature distribution near the focal point of the laser, COMSOL was utilized incorporating realistic material properties for water and glass. We note that the absorption coefficient of glass was 40 times that of water at the printing laser wavelength, which best matched experimental observations. This absorption coefficient is within the range of published values for glass. The COMSOL simulation utilized an axisymmetric model with a matched chamber height of 120 µm and a radius of ~ 60 µm, ensuring that the open boundaries didn't influence the results. The Matlab code is available upon request.

TiO_2 Nanocrystal Characterization:

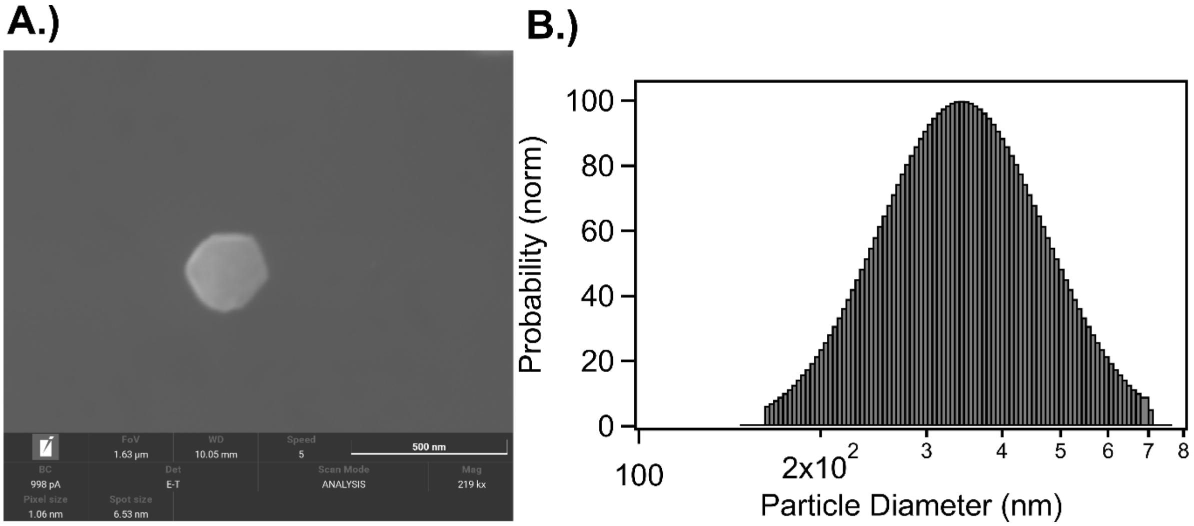

Figure S1: TiO_2 nanocrystal characterization. (A) SEM image of an individual TiO_2 nanocrystal. Scale bar is 500 nm. (B) DLS size distribution histogram of TiO_2 nanocrystals in water, showing an average particle diameter of 327.78 nm.

Error Measurement and Bootstrapping Analysis:

Each data point for accuracy in Figure 3B and Figure 5A was gathered by printing 25 particles in a 5 by 5 array as described in the experimental section. Particle positions were fit to a 5x5 grid, fitting a skew angle and distance between particles to account for minor experimental variations.

Actual position was subtracted from predicted position, which is denoted as positional error. The collective measure of accuracy was the standard deviation in positional error, which only returns one number per array. To give an estimate in the error of the measurement, we used bootstrapping. Briefly, for each data set of 25 particles, we randomly selected a subset. From this new set a standard deviation was calculated. We repeated this process to give a distribution of standard deviations possible given the original data set. In this case, this process was repeated 1,000,000 times to generate a bell-curve like plot, which gives an estimate of the upper and lower bound on standard deviation given the measurement. The 2.5% quantile and 97.5% quantile thresholds were used as the upper and lower bounds on standard deviation and are reported as error bars in the manuscript.

Multi-particle print event handling:

Occasionally, multiple particles will print at once on a printing location. These multi-particle print events are included in the final accuracy calculation. The distribution of multi-particle print events for the arrays presented in the main text are provided here as Figure S2.

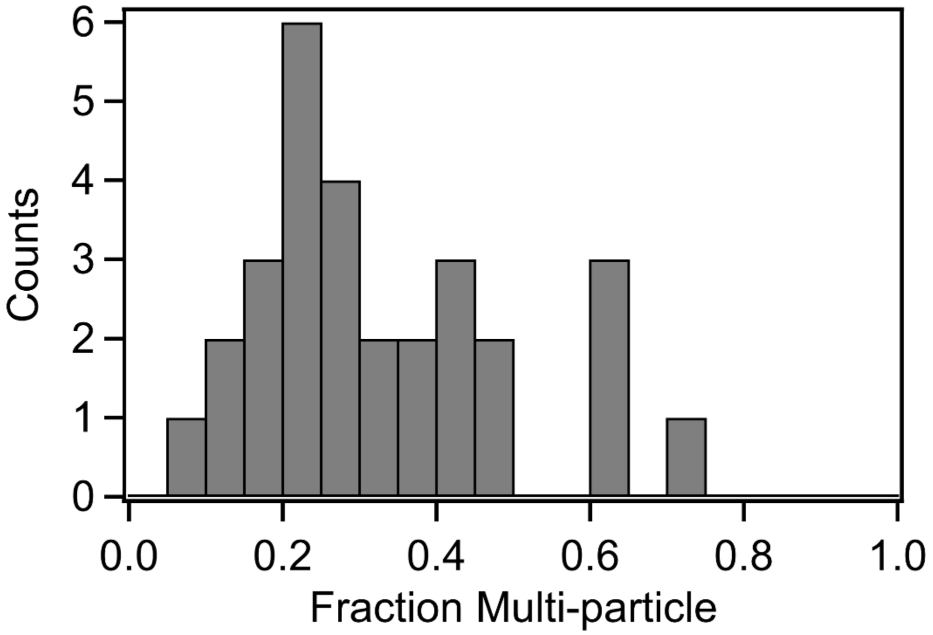

Figure S2: Histogram of fraction of printed particles that were multi-particle events (2 or more particles printed at a time) across arrays printed in the manuscript. The average fraction is 0.32 with a standard deviation of 0.17. No clear trends were observed between multi-particle prints and different experimental conditions. Thus the data and analysis presented in the manuscript are unaffected by multi-particle print events. Multi-particle print events are handled in the same way as single-particle print events, and are included in the σ_{position} measurements.

Simulation details and conditions:

To explain our experimental results, we constructed a Multiphysics simulation in Matlab combining thermal forces, DLVO theory, temperature-dependent viscosity, and kinetic energy of particles. There are multiple free parameters in this model that were chosen to match realistic values in literature or experimental observations. The following table lists each parameter with a description of what it is, its value, and source as necessary:

Parameter	Definition	Value	Citation
Electrolyte Concentration	Concentration of electrolyte in solution for DLVO theory calculations	For water, 1E-7 M	-
n_m	Refractive index of the media	1.33 (water)	[3]
n_p	Refractive index of the particle	2.6 (TiO_2)	[4]
λ	Wavelength of laser	976 nm	-
R_p	Particle radius	165 nm	-
NA	Numerical aperture of objective	1.3	-
$\rho_{Particle}$	Density of particle	4230 kg/m^3	[5]

ϵ_1	Static relative permittivity of particle (used in Hamaker calculation)	70	[6]
ϵ_2	Static relative permittivity of glass (used in Hamaker calculation)	7	[7]
ϵ_3	Static relative permittivity of water (used in Hamaker calculation)	80.1	[8]
$\zeta_{Potential}$	Zeta potential of particle	-35.65 mV	Experimental (DLS)
$\zeta_{Surface}$	Zeta potential of the non-functionalized surface	-32 mV	[9]

The physical phenomena that are incorporated into the simulation are detailed below, including the equations used to account for them:

COMSOL Modeling:

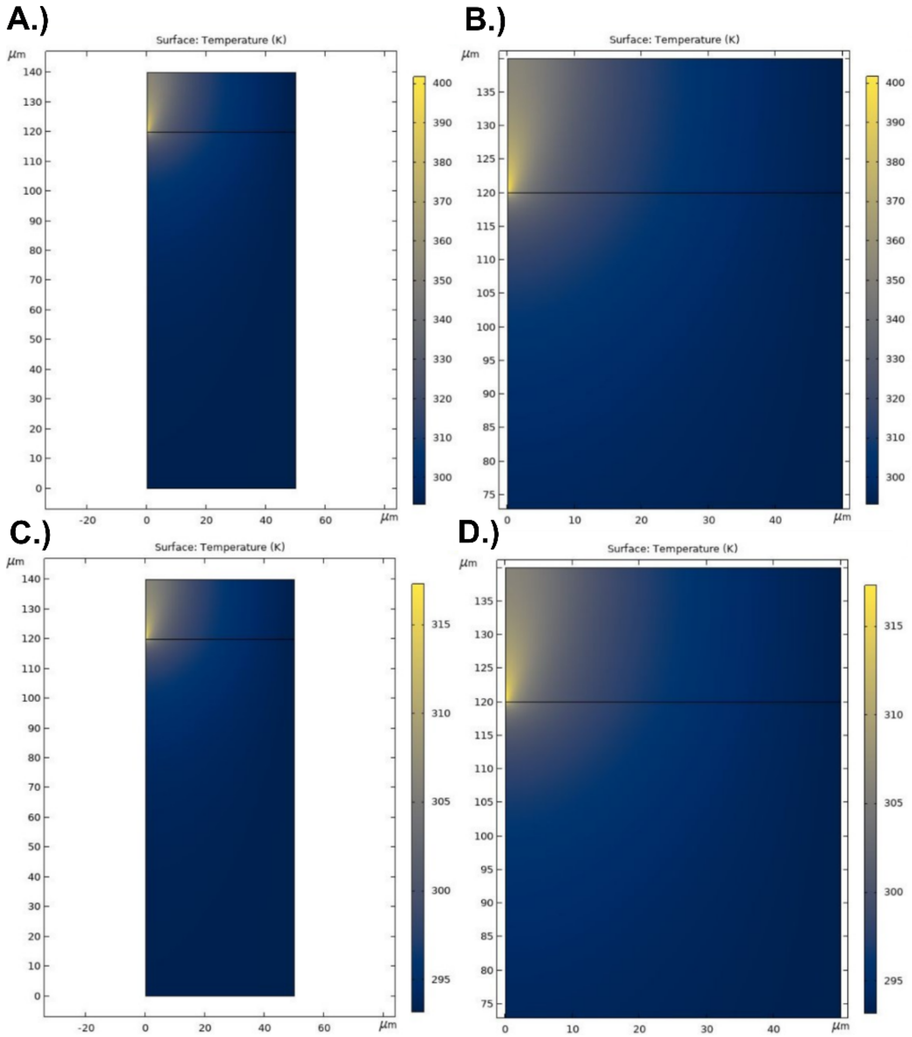

Figure S3: COMSOL modeled temperature profiles for high power (112 mW, panels A and B), and low power (24.6 mW, panels C and D) irradiation of a glass surface with water underneath it. At 112 mW, the maximum temperature increase in solution is ~97.5 K. At 24.6 mW, the maximum temperature increase in solution is ~22 K. The chamber consists of water with glass on top, where the absorption of glass is modeled to be 40 times that of water to create convection currents that were closer to the experimental value without pushing far past the boiling point of water.

Thermal convection:

Velocity distribution for fluid moving from thermal convection was extracted from COMSOL simulations of the chamber. COMSOL was used to model the temperature and convection velocity

profiles given the geometry of the chamber. The simulated temperature profile is shown in Figure S3. The largest temperature increase in solution is on the surface. The convection velocities calculated by COMSOL were compared to experimental videos of nanocrystals approaching and leaving the laser focus. Because these trajectories were outside the laser path and avoid printing, they provide a measurement of the convective velocity due to thermal fields rather than radiation pressure. The velocity of the nanocrystals leaving the laser focus was estimated from videos, and this is taken as the convection velocity at a location close to the focus. The COMSOL modelled convective velocities were scaled up by a factor of 2150 to match these experimental values. We recognize that the phenomena in the video could potentially be from a different chemical or physical effect driven by photothermal effects. Using a scaling factor incorporates these effects regardless of their origin.The effect of convective flow on particles was lumped into the drag force, where the velocity used is the relative velocity:

$$v_{rel}=v_{particle}-v_{convection}$$

Thermophoretic forces:

Thermophoresis of particles is modeled using the following equation:[10]

$$F_{TP}=S_T \frac{\Delta T}{\Delta x} k_B T$$

Where ΔT is the change in temperature between two points, Δx is the change in position between two points, k_B is the Boltzmann constant, T is the temperature, F_{TP} is thermophoretic force, and S_T is the Soret coefficient. The Soret coefficient for these particles is taken to be 0.18 K^{-1} noting that the exact value is difficult to measure or predict.[11] Using this value gives forces that are about a tenth of the thermal convection forces, and given the Soret coefficient matches that of polystyrene in literature this should be a reasonable estimate.

DLVO Theory:

DLVO force is calculated via the following equations[7]:

$$F_{DLVO} = F_{EDL} + F_{VDW}$$

Where electric double layer force is given by:

$$F_{EDL} = \kappa^{-1} r_P Z e^{-\kappa^{-1} h}$$

And Van der Waals forces are given by:

$$F_{VDW} = -\frac{H r_P}{6h^2}$$

Where κ^{-1} is inverse Debye length, r_P is particle radius, and h is distance between the particle and the surface. Z is a constant relating charges of the surface and particle for force calculation and is given by:

$$Z = 64\pi\epsilon_m\epsilon_0\left(\frac{k_B T}{C_e}\right)^2 \tanh\left(\frac{C_e\zeta_{particle}}{4k_B T}\right)\tanh\left(\frac{C_e\zeta_{surface}}{4k_B T}\right)$$

Where ϵ_m is the reduced electric permittivity of the solvent, ϵ_0 is vacuum permittivity, C_e is the fundamental electric charge, $\zeta_{particle}$ is the zeta potential of the particle and $\zeta_{surface}$ is the zeta potential of the surface. H is the Hamaker constant, given by:

$$H = 0.75k_B T\frac{\epsilon_1 - \epsilon_3}{\epsilon_1 + \epsilon_3}\frac{\epsilon_2 - \epsilon_3}{\epsilon_2 + \epsilon_3} + \frac{9E15\frac{h_P}{8\sqrt{2}}\left((n_1^2 - n_3^2)(n_2^2 - n_3^2)\right)}{(n_1^2 + n_3^2)^{0.5}(n_2^2 + n_3^2)^{0.5}((n_1^2 + n_3^2)^{0.5} + (n_2^2 + n_3^2)^{0.5})}$$

Where subscripts 1, 2, 3 represent the particle, surface, and media respectively. n is the refractive index, ϵ is the reduced electric permittivity, and h_P is Planck's constant. This gives a value on the order of 10^{-20} J, which matches the expected order of magnitude.

Viscosity:

Temperature dependent viscosity is taken to be based on a water model,[12] given by:

$$\log_{10}\frac{\mu}{\mu_0} = \frac{20 - T}{T + 96}(1.2378 - 1.303 \cdot 10^{-3}(20 - T) + 3.06 \cdot 10^{-6}\,(20 - T)^2 + 2.55 \cdot 10^{-8}(20 - T)^3)$$

Where μ is viscosity, μ_0 is viscosity at 20 °C, and T is temperature in °C.

Faxen's Correction:

When particles are close to a surface, viscosity is impacted by the boundary, and to correct this, a new viscosity can be calculated via the following relationships[13]:

$$\frac{\mu_{0,x}}{\mu_x} = (1 - \frac{9}{16}\frac{r_P}{h + r_P} + \frac{1}{8}\frac{r_P^3}{(h + r_P)^3})$$

Where $\mu_{0,x}$ is the unmodified viscosity in the x direction, μ_x is the new viscosity in the x direction, r_P is the particle radius, h is the distance from particle to the surface. For the z direction:

$$\frac{\mu_{0,z}}{\mu_z} = (1 - \frac{9}{8}\frac{r_P}{h + r_P} + \frac{1}{2}\frac{r_P^3}{(h + r_P)^3})$$

Combined equation of motion:

Combining all of the above to determine how the particle moves is performed by the following equation of motion:

$$a = \frac{F_{opt} + F_{DLVO} + F_{drag} + F_{TP}}{m}$$

Where a is the acceleration on the particle, F_{opt} is the optical force on the particle, F_{DLVO} is the DLVO force (only for the z direction), F_{TP} is the thermophoretic force, and m is the particle mass. F_{opt} is calculated by the Optical Tweezers Toolbox,[2] and drag force is calculated via Stokes' Law:

$$F_{drag} = 6\pi r_P \mu v_{rel}$$

Where r_P is particle radius, μ is viscosity (modified by Faxen's correction and temperature adjustments), v_{rel} is relative viscosity (from convective flow).

Acceleration is converted into position via:

$$a = \frac{d^2x}{dt^2}$$

Where finite differencing is used to solve for position as a function of time. The incremental time is chosen to ensure smooth movement with no artifacts.

Force of adhesion:

The force of adhesion can be roughly approximated for a sphere on a surface as[7]:

$$F_{adhesion} = 4\pi R W_{SL}$$

Where R is the radius of the sphere, and W_{SL} is the work of adhesion for the sphere to the surface mediated by the solvent. In this case, W_{SL} is a function of the surface tension of the fluid in contact with the surface. As the surface tension goes down, the W_{SL} term also goes down, causing a lower adhesion force, and therefore more attempts to print. This is shown by the 5% ethanol case in Figure 2A, where adding ethanol lowers the surface tension, causing many more print attempts to be required for identical irradiances.

Simulation without temperature dependence of viscosity:

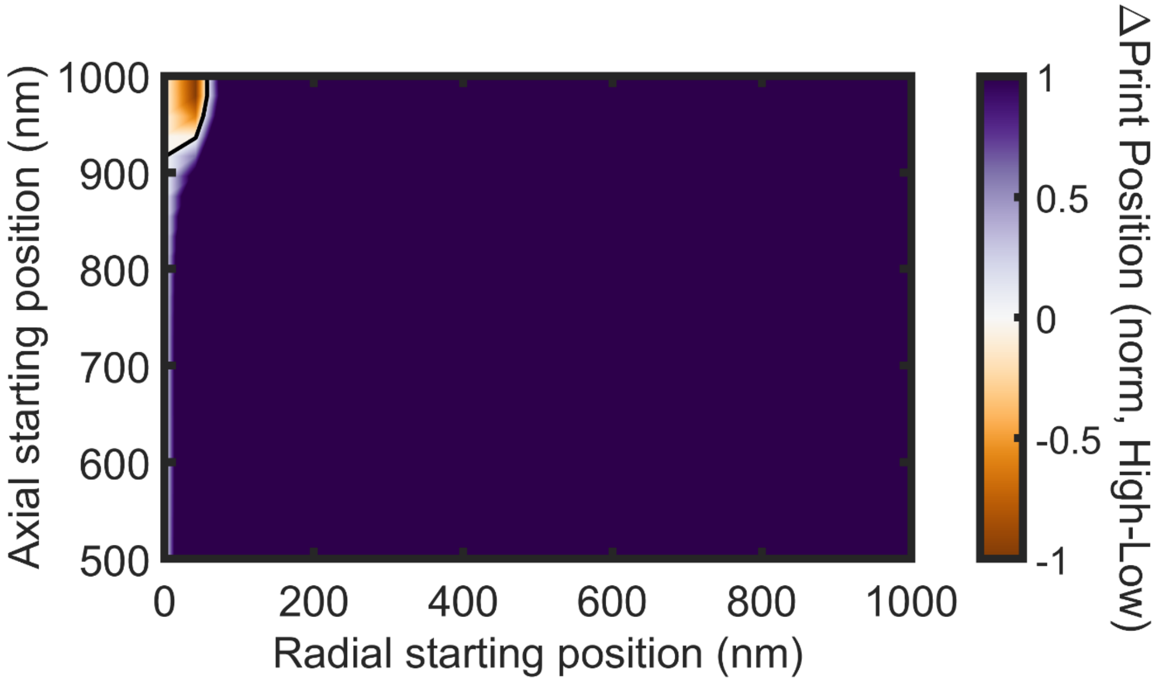

Figure S4. Multiphysics modeling of 300 nm TiO_2 being printed in water on glass. This simulation considers thermal forces as well as the mass of the particle but does not change viscosity with temperature. High power is 112 mW and low power is 24.6 mW both with a laser wavelength of 976 nm. This simulation is identical to Figure 4C, except that the viscosity of the solution is not a function of temperature. It can be seen that the behavior is lost in this case, indicating the importance of the reduction of viscosity with temperature in the behavior observed in Figure 3A.

DLVO theory energy barrier/force as a function of salt concentration and position:

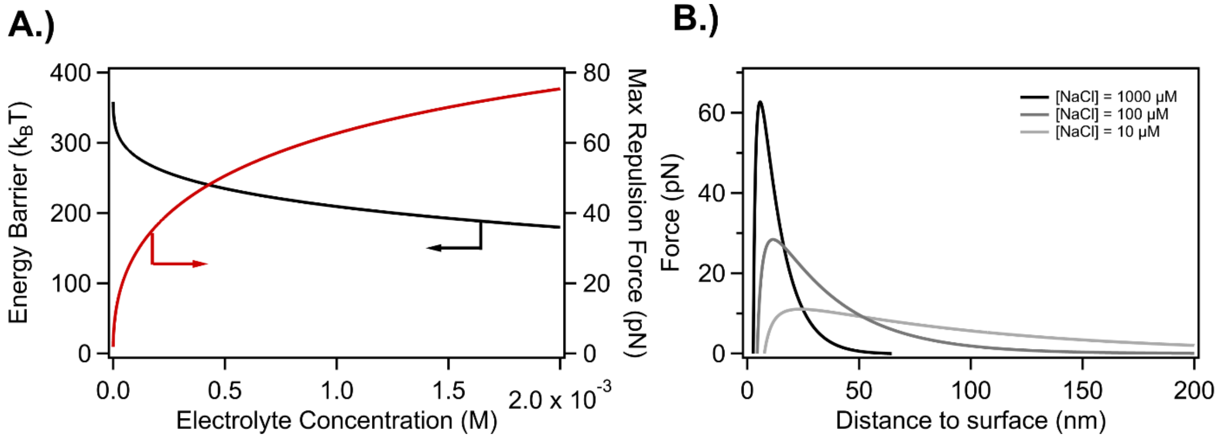

Figure S5. (A) Relationship between energy barrier (black line) and maximum repulsion force (red line) as a function of electrolyte concentration, in this case NaCl. As electrolyte concentration increases, the energy barrier decreases, but the maximum repulsion force increases to a point. This is a result of the compression of the DLVO interaction at higher electrolyte concentrations, leading to overall a reduced energy barrier, even if there is a greater maximum repulsion force. **(B)** DLVO repulsion force as a function of position for various electrolyte concentrations. Note that as electrolyte concentration increases, the interaction gets bunched closer to the surface.

Simulation behavior under high electrolyte concentration:

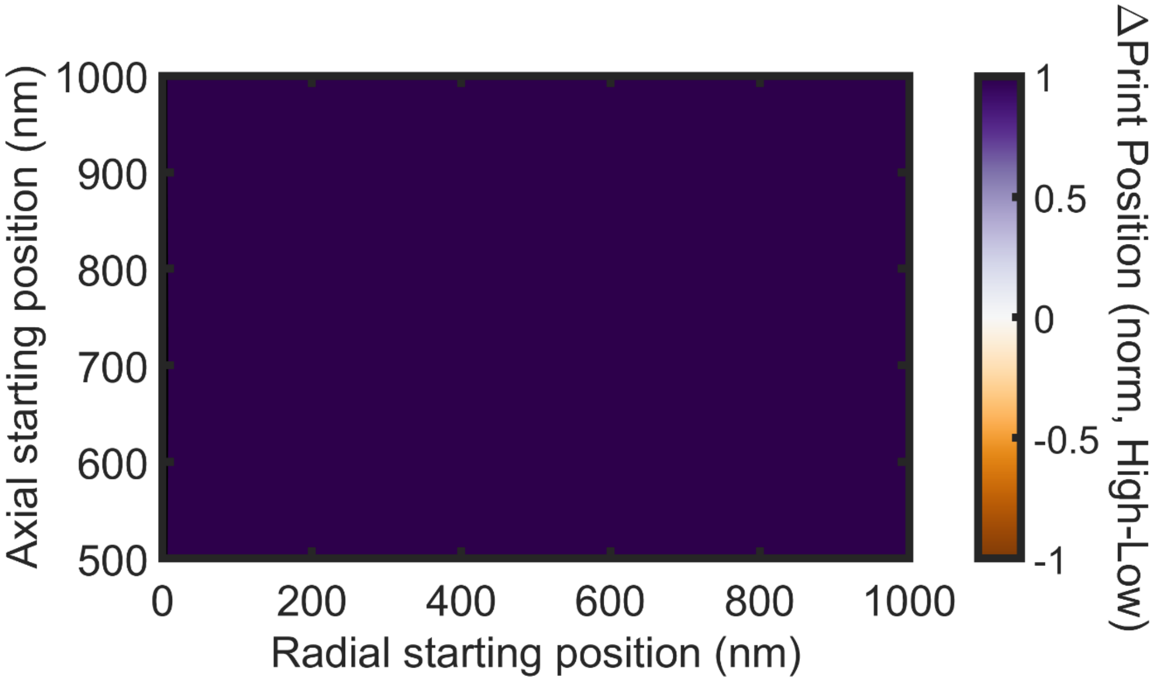

Figure S6. Multiphysics modeling of 300 nm TiO_2 being printed in water with 10 μM NaCl on glass. This simulation considers thermal forces as well as the mass of the particle. “High power” is 112 mW, “low power” is 24.6 mW with a laser wavelength of 976 nm. This simulation is identical to Figure 4C, except that the solution contains 10 μM NaCl, showing the behavior of accuracy improvement at high power is completely lost when salt concentration increases, matching the behavior of Figure 3A.

<u>Contact angle surface characterization for ITO and silica glass:</u>

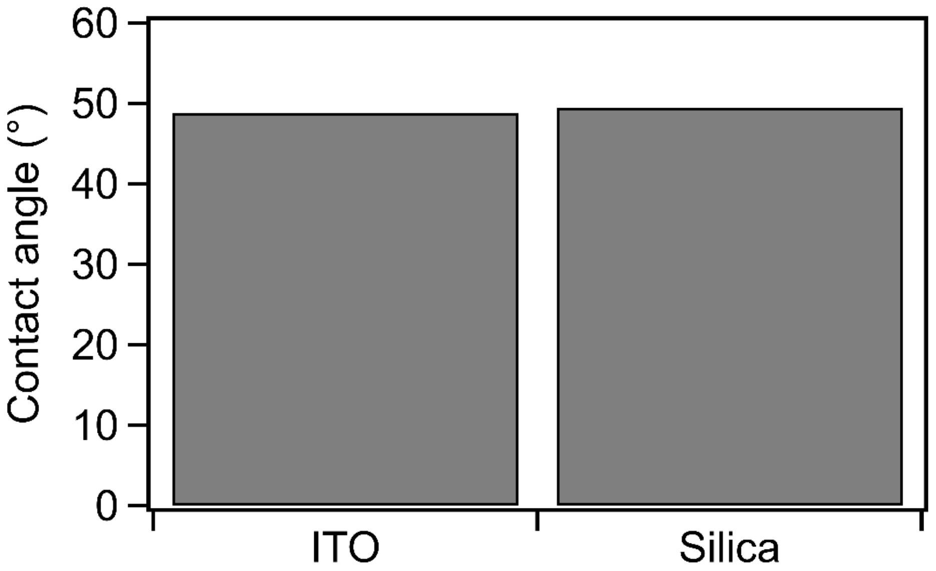

Figure S7. Contact angle measurement for ITO surface compared to silica glass surface, prepared in ways identical to those for printing experiments. The solution used in this case is identical to those used for Figure 5A, ITO was pure water, and silica was 2.6 mM NaCl solution. The contact angle is nearly identical, which suggests that under these conditions the surface interaction is comparable. Therefore, the trend noted in Figure 5A cannot be due to surface interactions alone and must be a result of the increased thermal interactions on an ITO surface.

Impact of pH on σ_{position} on glass:

Figure S8 shows the impact of pH on σ_{position} when printing TiO_2 nanocrystals on glass with a NaCl concentration of 5.1 mM. We observed that decreasing the pH increased the σ_{position}. Because decreasing the pH decreases the surface zeta potential, this experiment indicates that decreasing zeta potential decreases accuracy. This trend is consistent with our model, which predicts that lowering the zeta potential decreases the barrier to print. Literature values reliably show that the

isoelectric points of SiO_2, TiO_2, and ITO are ~ 2, 4, and 6 pH respectively, indicating that at neutral pH, the surface zeta potential is $SiO_2 > TiO_2 >$ ITO.[9,14–17] Thus, we would expect ITO to have a much higher σ_{position} compared to SiO_2. However, we observe the opposite. We interpret this improved σ_{position} as being due to photothermal effects.

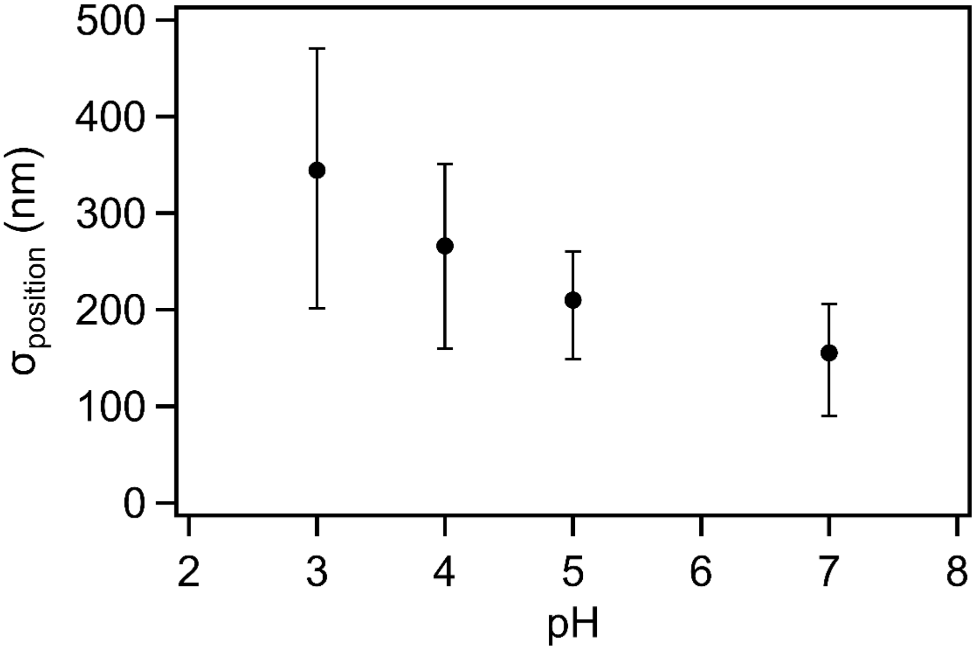

Figure S8. σ_{position} as a function of pH for printing TiO_2 nanocrystals on glass with a NaCl concentration of 5.1 mM. As pH decreases, σ_{position} is seen to increase. As pH decreases, the zeta potential of SiO_2 and TiO_2 is also known to decrease in magnitude.[9,18] This correlates a decrease in magnitude of zeta potential with an increase in magnitude of σ_{position}. This is consistent with literature, as a decrease in the magnitude of zeta potential indicates a lower repulsive force for the particle and surface. Laser irradiance was 14.3 MW/cm^2 for this experiment.

Experimental setup:

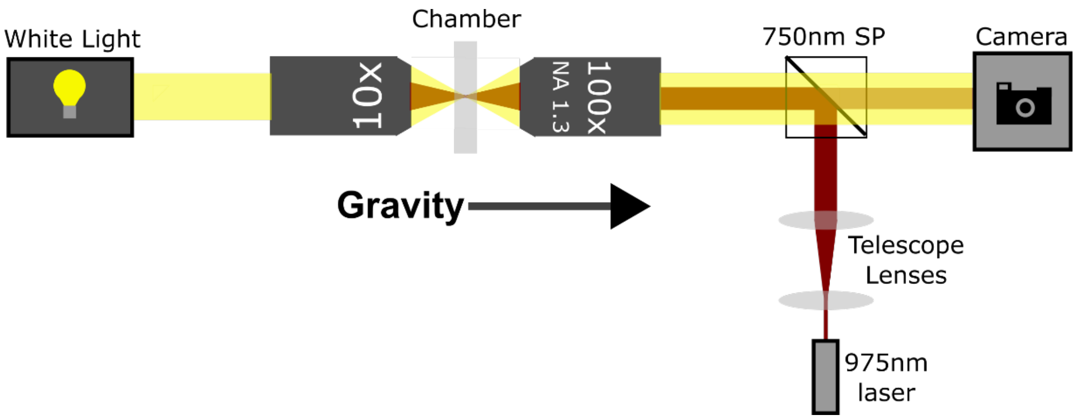

Figure S9. Cartoon diagram of optical printing setup. For printing, a 976-nm laser is expanded into a high numerical aperture (1.3) high magnification (100x) objective, where it enters through the sample chamber, and a particle is pressed against the top surface (left in this diagram) of the chamber, printing it in place. A piezo stage moves the chamber around, allowing different locations to be printed. White light is sent in via the 10x objective to view the printed particles in a transmission microscopy setup.

Supporting Information Citations:

(1) Reynolds, B.; Crane, M. J. Illuminating the Impact of Nonaqueous Solvents on Optical Trapping. *J. Phys. Chem. C* **2025**, *129* (20), 9405–9412. https://doi.org/10.1021/acs.jpcc.5c00640.

(2) Nieminen, T. A.; Loke, V. L. Y.; Stilgoe, A. B.; Knöner, G.; Brańczyk, A. M.; Heckenberg, N. R.; Rubinsztein-Dunlop, H. Optical Tweezers Computational Toolbox. *J. Opt. Pure Appl. Opt.* **2007**, *9* (8), S196. https://doi.org/10.1088/1464-4258/9/8/S12.

(3) Hale, G. M.; Querry, M. R. Optical Constants of Water in the 200-Nm to 200-Mm Wavelength Region. *Appl. Opt.* **1973**, *12* (3), 555–563. https://doi.org/10.1364/AO.12.000555.

(4) DeVore, J. R. Refractive Indices of Rutile and Sphalerite. *JOSA Vol 41 Issue 6 Pp 416-419* **1951**. https://doi.org/10.1364/JOSA.41.000416.

(5) Wypych, A.; Bobowska, I.; Tracz, M.; Opasinska, A.; Kadlubowski, S.; Krzywania-Kaliszewska, A.; Grobelny, J.; Wojciechowski, P. Dielectric Properties and Characterisation of Titanium Dioxide Obtained by Different Chemistry Methods. *J. Nanomater.* **2014**, *2014* (1), 124814. https://doi.org/10.1155/2014/124814.

(6) Takeuchi, M.; Itoh, T.; Nagasaka, H. Dielectric Properties of Sputtered TiO2 Films. *Thin Solid Films* **1978**, *51* (1), 83–88. https://doi.org/10.1016/0040-6090(78)90215-8.

(7) Israelachvili, J. N. *Intermolecular and Surface Forces*, 3rd ed.; Elsevier, 2011.
(8) Fernández, D. P.; Goodwin, A. R. H.; Lemmon, E. W.; Levelt Sengers, J. M. H.; Williams, R. C. A Formulation for the Static Permittivity of Water and Steam at Temperatures from 238 K to 873 K at Pressures up to 1200 MPa, Including Derivatives and Debye–Hückel Coefficients. *J. Phys. Chem. Ref. Data* **1997**, *26* (4), 1125–1166. https://doi.org/10.1063/1.555997.
(9) Bousse, L.; Mostarshed, S.; Van Der Shoot, B.; de Rooij, N. F.; Gimmel, P.; Göpel, W. Zeta Potential Measurements of Ta2O5 and SiO2 Thin Films. *J. Colloid Interface Sci.* **1991**, *147* (1), 22–32. https://doi.org/10.1016/0021-9797(91)90130-Z.
(10) Ide, K.; Tsuji, T.; Suzuki, T.; Setoura, K. Brownian Dynamics Simulation of Microscale Thermophoresis in Liquid. *ACS Omega* **2025**, *10* (5), 4526–4533. https://doi.org/10.1021/acsomega.4c08170.
(11) Duhr, S.; Braun, D. Why Molecules Move along a Temperature Gradient. *Proc. Natl. Acad. Sci.* **2006**, *103* (52), 19678–19682. https://doi.org/10.1073/pnas.0603873103.
(12) Kestin, J.; Sokolov, M.; Wakeham, W. A. Viscosity of Liquid Water in the Range −8 °C to 150 °C. *J. Phys. Chem. Ref. Data* **1978**, *7* (3), 941–948. https://doi.org/10.1063/1.555581.
(13) Leach, J.; Mushfique, H.; Keen, S.; Di Leonardo, R.; Ruocco, G.; Cooper, J. M.; Padgett, M. J. Comparison of Faxén's Correction for a Microsphere Translating or Rotating near a Surface. *Phys. Rev. E* **2009**, *79* (2), 026301. https://doi.org/10.1103/PhysRevE.79.026301.
(14) Usui, H.; Sasaki, T.; Koshizaki, N. Optical Transmittance of Indium Tin Oxide Nanoparticles Prepared by Laser-Induced Fragmentation in Water. *J. Phys. Chem. B* **2006**, *110* (26), 12890–12895. https://doi.org/10.1021/jp061866f.
(15) Liao, D. L.; Wu, G. S.; Liao, B. Q. Zeta Potential of Shape-Controlled TiO2 Nanoparticles with Surfactants. *Colloids Surf. Physicochem. Eng. Asp.* **2009**, *348* (1), 270–275. https://doi.org/10.1016/j.colsurfa.2009.07.036.
(16) Pan, R.; Pan, S.; Zhou, J.; Wu, Y. Surface-Modification of Indium Tin Oxide Nanoparticles with Titanium Dioxide by a Nonaqueous Process and Its Photocatalytic Properties. *Appl. Surf. Sci.* **2009**, *255* (6), 3642–3647. https://doi.org/10.1016/j.apsusc.2008.10.010.
(17) Suzuki, R.; Nishi, Y.; Matsubara, M.; Muramatsu, A.; Kanie, K. Single-Crystalline Protrusion-Rich Indium Tin Oxide Nanoparticles with Colloidal Stability in Water for Use in Sustainable Coatings. *ACS Appl. Nano Mater.* **2020**, *3* (5), 4870–4879. https://doi.org/10.1021/acsanm.0c01023.
(18) Choi, S. C.; Sohn, S. H. Synthesis and Physical Properties of TiO2 Microparticles Coated by a Sol–Gel Method and Their Application to Dye-Sensitized Solar Cells. *Powder Technol.* **2012**, *226*, 157–164. https://doi.org/10.1016/j.powtec.2012.04.037.
